# Supplementary material for: Structural basis of Stu2 recruitment to yeast kinetochores
Source: eLife. 2021 Feb 16;10:e65389. doi: 10.7554/eLife.65389 (PMC7909949; doi:10.7554/eLife.65389)
Supplement: Supplementary file 1. — All strains are derivatives of M3 (W303). [file elife-65389-supp1.docx]

**Supplementary File 1.** Strains used in this study.

All strains are derivatives of M3 (W303)

**Strain Relevant Genotype**

| M3 (W303) | *MAT***a** *ura3-1 leu2-3,112 his3-11 trp1-1 can1-100 ade2-1 bar1-1* |
| --- | --- |
| M35 | *MAT***a** *mad2∆::URA3* |
| M36 | *MAT***a** *mad3∆::URA3* |
| M619 | *MAT***a** *STU2-3HA-IAA7:KanMX his3::pGPD1-OsTIR1:HIS3 DSN1-6His-3Flag:URA3* |
| M622 | *MAT***a** *STU2-3HA-IAA7:KanMX his3::pGPD1-OsTIR1:HIS3 DSN1-6His-3Flag:URA3 leu2::pSTU2-STU2-3V5:LEU2* |
| M653 | *MAT***a** *STU2-3HA-IAA7:KanMX his3::pGPD1-OsTIR1:HIS3 DSN1-6His-3Flag:URA3 leu2::pSTU2-stu2(∆855-888)-3V5:LEU2* |
| M1153 | *MAT***a** *pMET-CDC20:TRP1 his3::pCUP1-GFP-LacI cenIII-lacOx128:TRP1 trp1::pGPD1-OsTIR1:TRP1 STU2-3HA-IAA7:KanMX* |
| M1154 | *MAT***a** *pMET-CDC20:TRP1 his3::pCUP1-GFP-LacI cenIII-lacOx128:TRP1 trp1::pGPD1-OsTIR1:TRP1 STU2-3HA-IAA7:KanMX leu2::pSTU2-STU2-3V5:LEU2* |
| M1375 | *MAT***a** *TOR1-1 fpr1∆::NatMX* |
| M1387 | *MAT***a** *TOR1-1 fpr1∆::NatMX STU2-FRB:His3MX DSN1-6His-3Flag:URA3* |
| M1422 | *MAT***a** *TOR1-1 fpr1∆::NatMX NUF2-FKBP12:His3MX* |
| M1428 | *MAT***a** *TOR1-1 fpr1∆::NatMX DSN1-6His-3Flag:URA3 STU2-FRB:His3MX NUF2-FKBP12:His3MX* |
| M1441 | *MAT***a** *STU2-3HA-IAA7:KanMX his3::pGPD1-OsTIR1:HIS3 DSN1-6His-3Flag:URA3 leu2::pSTU2-stu2(M876E)-3V5:LEU2* |
| M1442 | *MAT***a** *STU2-3HA-IAA7:KanMX his3::pGPD1-OsTIR1:HIS3 DSN1-6His-3Flag:URA3 leu2::pSTU2-stu2(I873E)-3V5:LEU2* |
| M1443 | *MAT***a** *STU2-3HA-IAA7:KanMX his3::pGPD1-OsTIR1:HIS3 DSN1-6His-3Flag:URA3 leu2::pSTU2-stu2(L869E)-3V5:LEU2* |
| M1444 | *MAT***a** *STU2-3HA-IAA7:KanMX his3::pGPD1-OsTIR1:HIS3 DSN1-6His-3Flag:URA3 leu2::pSTU2-stu2(M876E I873E L869E)-3V5:LEU2* |
| M1461 | *MAT***a** *TOR1-1 fpr1∆::NatMX DSN1-6His-3Flag:URA3 MPS1-FRB:KanMX NUF2-FKBP12:His3MX* |
| M1476 | *MAT***a** *TOR1-1 fpr1∆::NatMX DSN1-6His-3Flag:URA3 trp1::pGPD1-OsTIR1:TRP1 STU2-3HA-IAA7:KanMX* |
| M1505 | *MAT***a** *TOR1-1 fpr1∆::NatMX DSN1-6His-3Flag:URA3 trp1::pGPD1-OsTIR1:TRP1 STU2-3HA-IAA7:KanMX NUF2-FKBP12:His3MX leu2::pSTU2-STU2-FRB-3V5:LEU2* |
| M1507 | *MAT***a** *TOR1-1 fpr1∆::NatMX DSN1-6His-3Flag:URA3 trp1::pGPD1-OsTIR1:TRP1 STU2-3HA-IAA7:KanMX NUF2-FKBP12:His3MX leu2::pSTU2-stu2(M876E I873E L869E)-FRB-3V5:LEU2* |
| M1513 | *MAT***a** *TOR1-1 fpr1∆::NatMX DSN1-6His-3Flag:URA3 trp1::pGPD1-OsTIR1:TRP1 STU2-3HA-IAA7:KanMX leu2::pSTU2-STU2-FRB-3V5:LEU2* |
| M1515 | *MAT***a** *TOR1-1 fpr1∆::NatMX DSN1-6His-3Flag:URA3 trp1::pGPD1-OsTIR1:TRP1 STU2-3HA-IAA7:KanMX leu2::pSTU2-stu2(M876E I873E L869E)-FRB-3V5:LEU2* |
| M1525 | *MAT***a** *STU2-3HA-IAA7:KanMX his3::pGPD1-OsTIR1:HIS3 DSN1-6His-3Flag:URA3 leu2::pSTU2-stu2(M876A I873A L869A)-3V5:LEU2* |
| M1541 | *MAT***a** *STU2-3HA-IAA7:KanMX his3::pGPD1-OsTIR1:HIS3 DSN1-6His-3Flag:URA3 leu2::pSTU2-stu2(M876E I873E L869E)-3V5:LEU2 mad3∆::NatMX* |
| M1554 | *MAT***a** *TOR1-1 fpr1∆::NatMX DSN1-6His-3Flag:URA3 trp1::pGPD1-OsTIR1:TRP1 STU2-3HA-IAA7:KanMX NUF2-FKBP12:His3MX leu2::pSTU2-stu2(∆855-888)-FRB-3V5:LEU2* |
| M1574 | *MAT***a** *STU2-3HA-IAA7:KanMX his3::pGPD1-OsTIR1:HIS3 DSN1-6His-3Flag:URA3 leu2::pSTU2-stu2(M876A)-3V5:LEU2* |
| M1575 | *MAT***a** *STU2-3HA-IAA7:KanMX his3::pGPD1-OsTIR1:HIS3 DSN1-6His-3Flag:URA3 leu2::pSTU2-stu2(I873A)-3V5:LEU2* |
| M1576 | *MAT***a** *STU2-3HA-IAA7:KanMX his3::pGPD1-OsTIR1:HIS3 DSN1-6His-3Flag:URA3 leu2::pSTU2-stu2(L869A)-3V5:LEU2* |
| M1577 | *MAT***a** *STU2-3HA-IAA7:KanMX his3::pGPD1-OsTIR1:HIS3 DSN1-6His-3Flag:URA3 leu2::pSTU2-stu2(M876A I873A L869A)-3V5:LEU2* |
| M1587 | *MAT***a** *TOR1-1 fpr1∆::NatMX DSN1-6His-3Flag:URA3 trp1::pGPD1-OsTIR1:TRP1 STU2-3HA-IAA7:KanMX leu2::pSTU2-stu2(∆855-888)-FRB-3V5:LEU2* |
| M1610 | *MAT***a** *pMET-CDC20:TRP1 his3::pCUP1-GFP-LacI cenIII-lacOx128:TRP1 trp1::pGPD1-OsTIR1:TRP1 STU2-3HA-IAA7:KanMX leu2::pSTU2-stu2(M876E I873E L869E)-3V5:LEU2* |
| M1622 | *MAT***a** *STU2-3HA-IAA7:KanMX his3::pGPD1-OsTIR1:HIS3 DSN1-6His-3Flag:URA3 leu2::pSTU2-STU2-3V5:LEU2 mad3∆::NatMX* |
| M1714 | *MAT***a***/MATɑ STU2/stu2∆::His3MX leu2::pSTU2-stu2(L869E I873E M876E)-3V5:LEU2/leu2::pSTU2-stu2(L869E I873E M876E)-3V5:LEU2* |
| M1716 | *MAT***a***/MATɑ STU2/stu2∆::His3MX leu2::pSTU2-stu2(∆855-888)-3V5:LEU2/leu2::pSTU2-stu2(∆855-888)-3V5:LEU2* |
| M1718 | *MAT***a***/MATɑ STU2/stu2∆::His3MX leu2::pSTU2-STU2-3V5:LEU2/leu2::pSTU2-STU2-3V5:LEU2* |
| M1757 | *MAT***a** *STU2-3HA-IAA7:KanMX CDC20-IAA17:KanMX trp1::pGPD1-OsTIR1:TRP1 ura3::CFP-TUB1:URA3 SPC110-mCherry:HPHMX leu2::pSTU2-STU2-GFP:LEU2* |
| M1761 | *MAT***a** *STU2-3HA-IAA7:KanMX CDC20-IAA17:KanMX trp1::pGPD1-OsTIR1:TRP1 ura3::CFP-TUB1:URA3 SPC110-mCherry:HPHMX leu2::pSTU2-stu2(∆855-888)-GFP:LEU2* |
| M1933 | *MAT***a** *STU2-3HA-IAA7:KanMX his3::pGPD1-OsTIR1:HIS3 DSN1-6His-3Flag:URA3 leu2::pSTU2- stu2(M876E I873E L869E)-3V5:LEU2 mad2∆::URA3* |
| M1985 | *MAT***a** *STU2-3HA-IAA7:KanMX CDC20-IAA17:KanMX trp1::pGPD1-OsTIR1:TRP1 ura3::CFP-TUB1:URA3 SPC110-mCherry:HPHMX leu2::pSTU2-stu2(M876E I873E L869E)-GFP:LEU2* |
| M2024 | *MAT***a** *TOR1-1 fpr1∆::NatMX his3::pCUP1-GFP-LacI cenIII-lacOx128:TRP1 trp1::pGPD1-OsTIR1:TRP1 STU2-3HA-IAA7:KanMX mad3::HPHMX leu2::pSTU2-stu2(M876E I873E L869E)-FRB-3V5:LEU2* |
| M2025 | *MAT***a** *TOR1-1 fpr1∆::NatMX his3::pCUP1-GFP-LacI cenIII-lacOx128:TRP1 trp1::pGPD1-OsTIR1:TRP1 STU2-3HA-IAA7:KanMX mad3::HPHMX leu2::pSTU2-STU2-FRB-3V5:LEU2* |
| M2026 | *MAT***a** *TOR1-1 fpr1∆::NatMX his3::pCUP1-GFP-LacI cenIII-lacOx128:TRP1 trp1::pGPD1-OsTIR1:TRP1 STU2-3HA-IAA7:KanMX mad3::HPHMX NUF2-FKBP12:His3MX leu2::pSTU2-stu2(M876E I873E L869E)-FRB-3V5:LEU2* |
| M2027 | *MAT***a** *TOR1-1 fpr1∆::NatMX his3::pCUP1-GFP-LacI cenIII-lacOx128:TRP1 trp1::pGPD1-OsTIR1:TRP1 STU2-3HA-IAA7:KanMX mad3::HPHMX NUF2-FKBP12:His3MX leu2::pSTU2-STU2-FRB-3V5:LEU2* |
| M2029 | *MAT***a** *pMET-CDC20:TRP1 his3::pCUP1-GFP-LacI cenIII-lacOx128:TRP1 trp1::pGPD1-OsTIR1:TRP1 STU2-3HA-IAA7:KanMX ura3::pSPC110-SPC110-mCherry:URA3 leu2::pSTU2-STU2-3V5:LEU2* |
| M2030 | *MAT***a** *pMET-CDC20:TRP1 his3::pCUP1-GFP-LacI cenIII-lacOx128:TRP1 trp1::pGPD1-OsTIR1:TRP1 STU2-3HA-IAA7:KanMX ura3::pSPC110-SPC110-mCherry:URA3 leu2::pSTU2-stu2(M876E I873E L869E)-3V5:LEU2* |
