# Supplementary material for: Structural basis of Stu2 recruitment to yeast kinetochores
Source: eLife. 2021 Feb 16;10:e65389. doi: 10.7554/eLife.65389 (PMC7909949; doi:10.7554/eLife.65389)
Supplement: Supplementary file 3. [file elife-65389-supp3.docx]

|  | **Stu2 CTS bound to dwarf Ndc80c*** |
| --- | --- |
| **Wavelength** | 0.97918 Å |
| **Resolution range** | 42.72 - 2.72 (2.817 - 2.72) |
| **Space group** | C 2 2 21 |
| **Unit cell** | 170.886 183.175 124.317 90 90 90 |
| **Total reflections** | 697683 (62919) |
| **Unique reflections** | 52106 (4719) |
| **Multiplicity** | 13.4 (13.3) |
| **Completeness (%)** | 99.03 (91.15) |
| **Mean I/sigma(I)** | 18.40 (1.78) |
| **Wilson B-factor** | 63.68 |
| **R-merge** | 0.1071 (0.9043) |
| **R-meas** | 0.1114 (0.9395) |
| **R-pim** | 0.03047 (0.2529) |
| **CC1/2** | 0.999 (0.829) |
| **CC*** | 1 (0.952) |
| **Reflections used in refinement** | 52103 (4719) |
| **Reflections used for R-free** | 2013 (194) |
| **R-work** | 0.1976 (0.2789) |
| **R-free** | 0.2416 (0.2910) |
| **CC(work)** | 0.962 (0.822) |
| **CC(free)** | 0.960 (0.757) |
| **Number of non-hydrogen atoms** | 5994 |
| **macromolecules** | 5869 |
| **ligands** | 30 |
| **solvent** | 95 |

**Supplementary File 3. Data collection and refinement statistics.**

| **Protein residues** | 713 |
| --- | --- |
| **RMS(bonds)** | 0.005 |
| **RMS(angles)** | 0.74 |
| **Ramachandran favored (%)** | 97.57 |
| **Ramachandran allowed (%)** | 2.43 |
| **Ramachandran outliers (%)** | 0.00 |
| **Rotamer outliers (%)** | 0.15 |
| **Clashscore** | 6.46 |
| **Average B-factor** | 88.87 |
| **macromolecules** | 89.23 |
| **ligands** | 89.23 |
| **solvent** | 63.61 |
| **Number of TLS groups** | 1 |

Statistics for the highest-resolution shell are shown in parentheses.
